# Reactive silver inks for antiviral, repellent medical textiles with ultrasonic bleach washing durability compared to silver nanoparticles

**DOI:** 10.1371/journal.pone.0270718

**Published:** 2022-09-14

**Authors:** Anthony J. Galante, Brady C. Pilsbury, Kathleen A. Yates, Melbs LeMieux, Daniel J. Bain, Robert M. Q. Shanks, Eric G. Romanowski, Paul W. Leu

**Affiliations:** 1 Department of Industrial Engineering, University of Pittsburgh, Pittsburgh, PA, United States of America; 2 Department of Ophthalmology, Charles T. Campbell Laboratory for Ophthalmic Microbiology, University of Pittsburgh School of Medicine, Pittsburgh, PA, United States of America; 3 Electroninks Inc, Austin, TX, United States of America; 4 Department of Geology and Environmental Science, University of Pittsburgh, Pittsburgh, PA, United States of America; University of Queensland, AUSTRALIA

## Abstract

Medical textiles are subject to particularly harsh disinfection procedures in healthcare settings where exposure risks are high. This work demonstrates a fabric treatment consisting of a reactive silver ink and low surface energy PDMS polymer that provides for superhydrophobicity and antiviral properties against enveloped herpes simplex virus stocks even after extended ultrasonic bleach washing. The antiviral properties of reactive silver ink has not been previously reported or compared with silver nanoparticles. The fabric treatment exhibits high static contact angles and low contact angle hysteresis with water, even after 300 minutes of ultrasonic bleach washing. Similarly, after this bleach washing treatment, the fabric treatment shows reductions of infectious virus quantities by about 2 logs compared to controls for enveloped viruses. The use of silver ink provides for better antiviral efficacy and durability compared to silver nanoparticles due to the use of reactive ionic silver, which demonstrates more conformal coverage of fabric microfibers and better adhesion. This study provides insights for improving the wash durability of antiviral silver fabric treatments and demonstrates a bleach wash durable, repellent antiviral treatment for reusable, functional personal protective equipment applications.

## Introduction

Personal protective equipment (PPE) such as gowns, masks, and scrubs are essential for protecting healthcare professionals from contact with bacteria and viruses that lead to infection. There is a need for textile coatings that provide PPE with better protection from infection by not only repelling fluids such as respiratory droplets, but also killing or deactivating microbes [1]. In particular, there is great interest in fabric coatings that provide these protective functionalities in PPE even after repeated use and laundering [2–4]. Furthermore, there is need for new fabric coatings that can withstand particularly harsh disinfection procedures such as high concentrations of bleach and ultrasonic cleaning that may be used in high risk settings such as healthcare. This work demonstrates a reactive silver ink based fabric treatment that offers longer lasting antiviral properties compared to silver nanoparticles after ultrasonic bleach washing. The antiviral properties of reactive silver ink has not been previously reported or compared with silver nanoparticles.

There has been a tremendous amount of research on the use of silver nanoparticles as an antimicrobial agent [5, 6] and their incorporation into a range of fabrics or masks [7–13]. Silver nanoparticles have been shown to inhibit the growth of yeast, *Escherichia coli*, and *Staphylococcus aureus* [14]. More recently, silver nanoparticles have been demonstrated to have antiviral properties against enveloped viruses such as severe acute respiratory syndrome coronavirus 2 (SARS-CoV-2) and human immunodeficiency viruses (HIV) [15–18].

However, nanoparticles on fabrics may have issues with being removed in the wash, resulting in the loss of any associated functionality [19, 20]. Recently published articles fail to investigate the washing durability of silver nanoparticles incorporated into fabrics for the use of personal protective equipment [10, 12]. Washed out nanoparticles may pose a threat to aquatic organisms [21–24] and bioaccumulate in the food chain [25]. Also, the efficacy of silver nanoparticles may be hindered after washing with bleach as most commonly encountered bleaches tend to be oxidising [5, 20]. Silver nanoparticles have poor adhesion to the underlying textile fibers due to the small contact area and weak van der Waals forces with the textiles. Significant research has been dedicated to improving the adhesion forces of silver nanoparticles with textile fibers such as the use of polymer coatings [26], polymer binders [27], binders [12, 28], silanization [29], in-situ reduction [13] and nanoparticle/polymer composites [30]. Nevertheless, nanoparticle-based methods are inherently limited by the non-uniformity of the particle deposition and difficulties in controlling silver release [31, 32].

There has been no previous work on the antiviral properties of silver fabric treatments after disinfection with bleach (sodium hypochlorite) concentrations as high as 10% and ultrasonic cleaning. Bleach (sodium hypochlorite) is a strong oxidant that is chemically reactive with organic molecules [33, 34]. The Centers for Disease Control and Prevention (CDC) recommends cleaning and disinfecting surfaces with a bleach washing concentration up to 5,000 ppm (1:10 dilution of household bleach) [35]. Reusable textiles in healthcare settings may similarly be decontaminated by washing in bleach solutions as high as 10% [35, 36]; therefore, it is essential to study the bleach wash durability of antimicrobial/antiviral techniques for medical fabric applications. This article utilizes the highest bleach concentration recommended by the CDC in the bleach washing procedure to ensure the potential for reusability in medical settings. Furthermore, ultrasonic cleaning induces cavitation bubbles to produce high mechanical forces on the fabric [37] and mechanical stress has been shown to play a dominant role in the removal of silver from fabrics [31]. This paper evaluates fabrics cleaned by ultrasonic cleaning (under ASTM G131–96 standards), which has been shown to achieve better detergency [38].

This work studies the virucidal activity of single knit polyethylene terephthalate (PET) fabrics coated with a silver amine complex ink [39] against herpes simplex viruses (HSV-1). This is the first report in literature where reactive silver inks have been considered in developing antiviral medical fabrics. This work investigates and discusses the advantages of silver inks compared to silver nanoparticles for reusable antiviral medical fabrics. PET fabric material is commonly used for medical and healthcare applications such as gowns, scrubs and caps due to its low cost, elasticity and durability [40]. HSV-1 is an enveloped virus about 160 nm in diameter that can cause cold sores and blinding herpetic eye infections [41]. The attachment and entry of HSV-1 into cells require the interaction between the viral envelope glycoproteins and cell surface heparan sulfate (HS) [42, 43]. Silver mimics HS and competes for binding sites of the virus to the cell, inhibiting the virus from cell attachment and entry [42, 44].

The reactive silver ink demonstrates better antiviral and bleach wash durability compared to 20 nm diameter silver nanoparticles. The size of the silver nanoparticles is considered as it is the maximum size to offer virus inhibition with HSV-1 and HIV-1 [17, 43]. Also, silver nanoparticles of similar size are shown to inhibit human coronavirus on mask fabric [12].

The chemical reduction of reactive silver ink into a pure silver film is previously described [39]. The reactive silver ink reduces *in-situ* on the textile and uniformly covers the microfibers of the fabric. This provides for better silver adhesion and uniformity with the fabric microfibers compared to nanoparticles, which adhere to the fabric with weak van der Waals forces. PET fabrics coated with 20 nm silver nanoparticles show a 1.1±0.6 log_10_ (92.4%, *p* < 0.05) log reduction in virus quantities compared to control fabric, while PET fabrics coated with reactive silver inks reduce virus quantities by 2.3 ± 0.8 log_10_ (99.5%, *p* < 0.001).

However, the results suggest the performance of both silver fabric treatments can be significantly hindered after extended periods of ultrasonic bleach washing. An additional polydimethylsiloxane (PDMS) thin film is used protect the silver layer and increase the liquid repellent properties of the fabric to further improve the bleach wash durability of the antiviral functionality. When the silver layer is protected by the polymer, the silver ink treated fabric retains 42% more silver than silver nanoparticle treated fabrics after bleach washing. PET fabric samples coated with reactive silver ink and PDMS reduce virus quantities by 1.7 ± 0.2 log_10_ (98.2%, *p* < 0.01) after 300 minutes of ultrasonic bleach washing. We demonstrate the combination of reactive silver ink with PDMS as a PET fabric treatment that adds durable, superhydrophobic and antiviral properties for reusable PPE applications.

## Results and discussion

**Fig 1:**
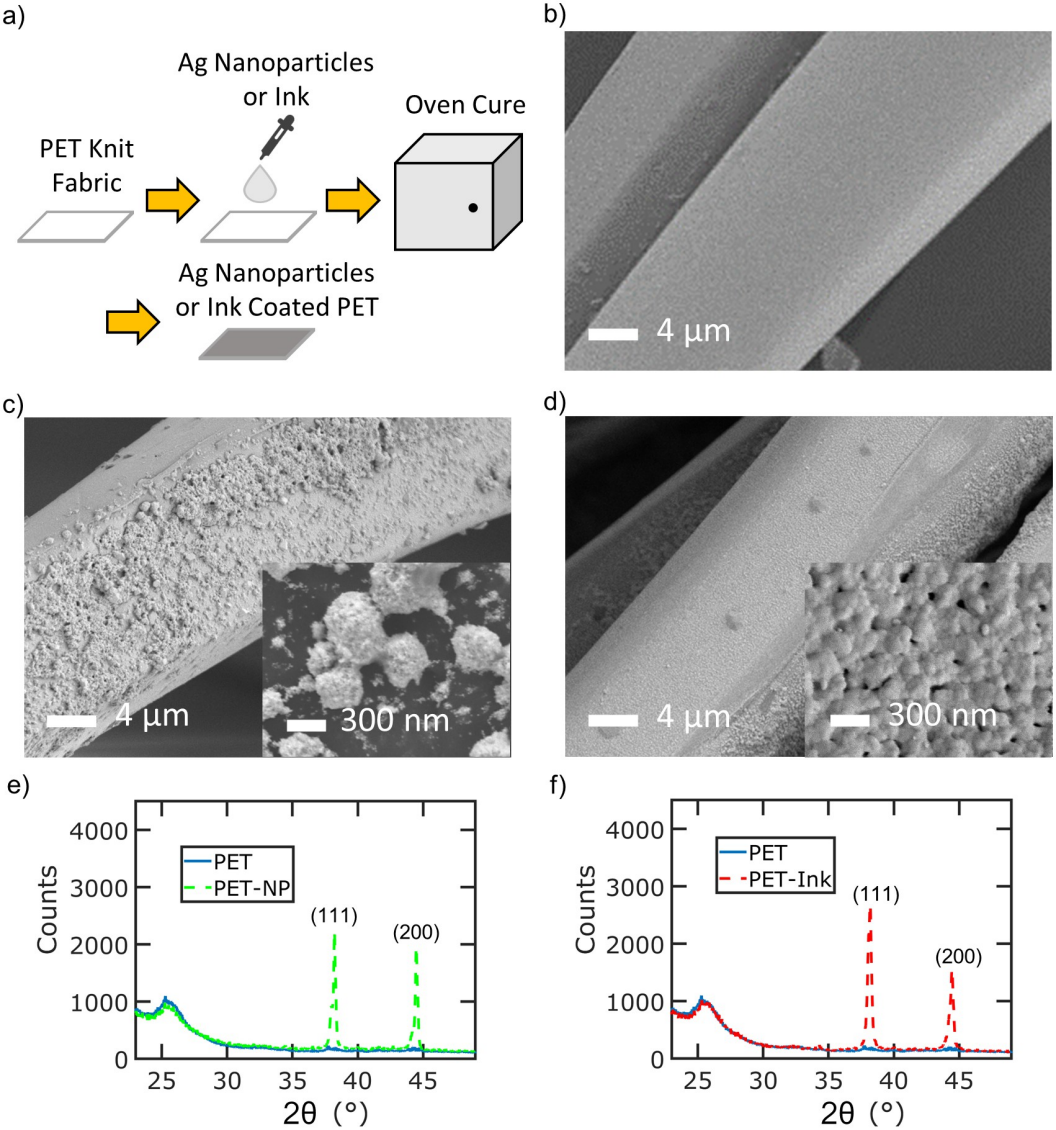
Sample characterization of silver coated fabric. a) Schematic of silver treatment on PET fabric (b-d) SEM images of b) untreated PET c) PET-NP and d) PET-Ink. e-f) XRD pattern of e) PET-NP and f) PET-Ink samples.

Fig 1 highlights the characterization of PET fabric after coating with silver nanoparticles or reactive silver ink. The PET fabrics are coated with silver by drop casting and then thermally curing in an oven (Fig 1a). Square fabric samples of 0.5” by 0.5” are coated with either silver nanoparticles or silver ink by drop casting, followed by curing in an oven for 1 hour at 120°C. An equal amount of silver by weight (0.2 mg) was added to each fabric. Untreated PET knit fabric consists of microfibers about 12–15 μm in diameter (Fig 1b). PET fabrics coated with 20 nm silver nanoparticles (PET-NP) show aggregation and non-uniform attachment over the surface of the PET (Fig 1c). In contrast, PET fabrics coated with silver ink (PET-Ink) show more uniform coverage around the microfibers of the fabric (Fig 1d). X-ray diffraction (XRD) is performed on the coated samples (Fig 1e and 1f). Distinct peaks are observed at 2*θ* = 38.2° and 44.5° corresponding to the (111) and (200) crystal planes. The XRD analysis confirms the presence of poly-crystalline on coated PET with silver nanoparticles (Fig 1e) or reactive silver ink (Fig 1f). The XRD characterization also shows the silver is not oxidized on the fabric after curing.

**Fig 2:**
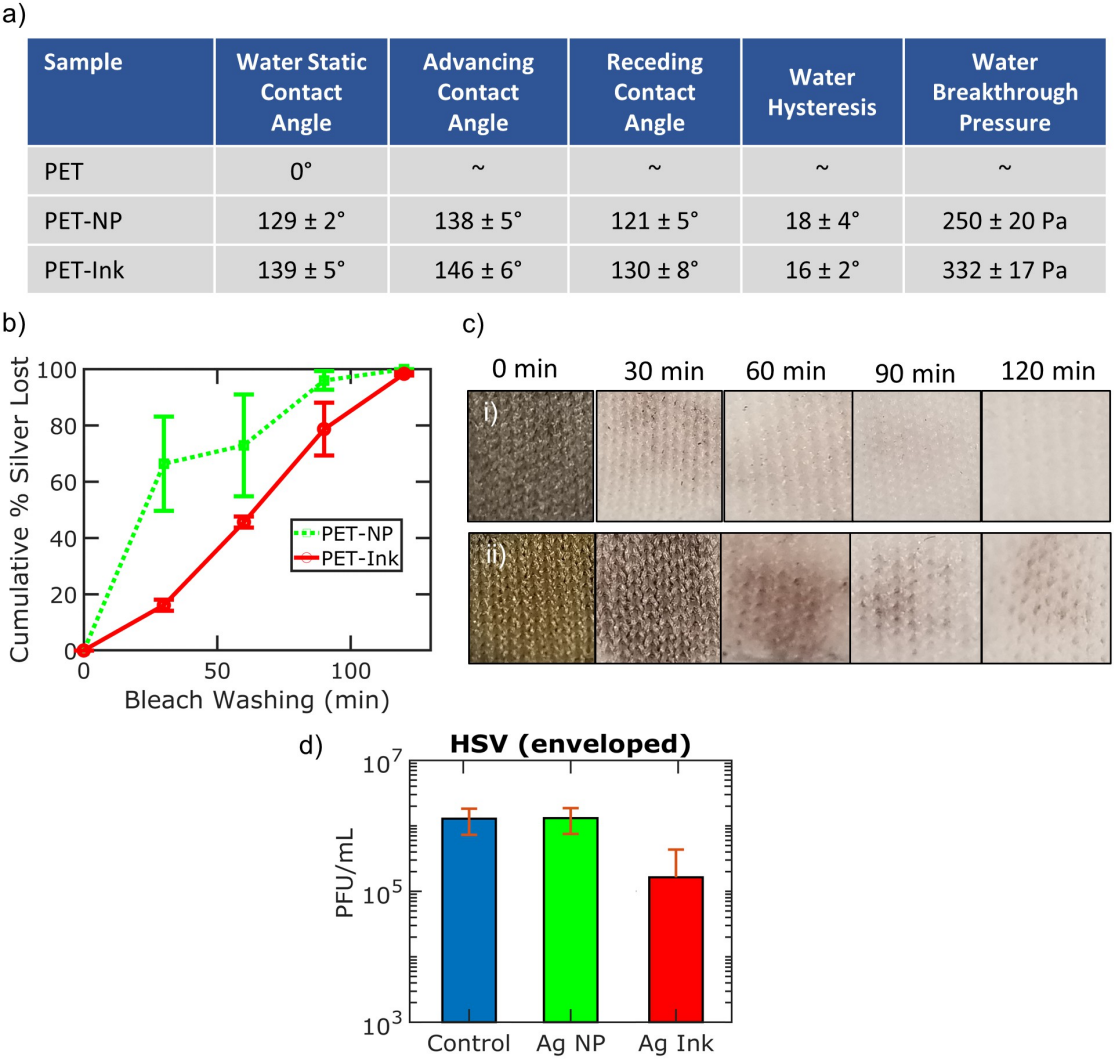
Wash durability of silver coated fabric. a) Wetting characterization for PET, PET-NP, and PET-Ink samples. b) Silver leaching analysis as a function of bleach washing time c) Optical images of (i) PET-NP and (ii) PET-Ink samples as a function of bleach washing time. d) Virus inhibition assay results of silver treatments in solution.

Fig 2 summarizes the wetting properties and bleach wash durability of coated PET samples, as well as the virus inhibition properties of the silver coatings in solution. Fig 2a shows the wetting properties of PET samples with water. Untreated PET is fully wetted by water. The water static contact angle and hysteresis of PET-NP are 129 ± 2° and 18 ± 4°, respectively. The breakthrough pressure is estimated to be 250 ± 20 Pa based on the water droplet radius when the droplet transitions from a Cassie-Baxter state to a Wenzel state. The water static contact angle and hysteresis of PET-Ink are 139 ± 5° and 16 ± 2°, respectively. The breakthrough pressure is estimated to be 332 ± 17 Pa. Both coated PET samples show similar hydrophobic wetting properties due to the additional nanoscale roughness on the microfibers from the coatings.

Ultrasonic bleach washing experiments are performed to assess the durability of silver. The cumulative percentage of silver that comes off coated samples during bleach washing is shown in Fig 2b. The silver concentration from the affluent of each 30 minute washing cycle is measured using inductively coupled plasma mass spectrometry (ICP-MS). PET-NP samples lose 62 ± 9% of the coated silver after the first 30 minutes of washing. PET-Ink samples lose silver at a slower rate due to the more uniform coating and stronger attachment to the PET from the silver ink diamine complex. Both treatments lose at least 99% of the silver by 120 minutes of ultrasonic bleach washing. Fig 2c shows representative images of the (i) PET-NP and (ii) PET-Ink samples as a function of bleach washing cycles. The combination of ultrasonic agitation and bleach not only mechanically removes silver from the textile, but also chemically reacts with silver. It is evident that silver treated fabrics need additional surface treatment for reuse in bleach washing procedures.

Additonally, we investigate the anti-viral activity of the silver coatings in solution (Fig 2c). Virus assays in saline are conducted by mixing silver ink or silver nanoparticles (0.1%) in phosphate buffered saline (PBS) with HSV-1 in PBS for 1 hour, followed by centrifuging, removing the supernatant, and quantifying the amount of remaining virus using plaque forming units (PFU). Fig 2d shows the virus inactivation results of silver nanoparticles and silver reactive ink with enveloped HSV-1 in PBS at a concentration of 1 mg per mL (0.1%). 20 nm silver nanoparticle coating does not show HSV-1 inactivation with a 0.0 ± 0.1 log_10_ difference, while the silver ink shows HSV-1 inactivation in saline by 1.6 ± 0.9 log_10_ (97.3%) difference. Previous reports have suggested that virus inactivation is stronger with smaller diameter silver nanoparticles (≤ 15 nm) and it is possible the nanoparticles are too large to cover the binding sites of HSV-1 to inhibit cell entry [15]. In contrast, the reactive silver ink consists of reactive ionic silver which can form molecular clusters (< 3 nm) that may be able to better coat the binding sites of HSV and thus, provide for more virus inactivation.

A PDMS post treatment is added to the silver coated samples to improve the ultrasonic bleach wash durability. PDMS is a low surface energy polymer that offers liquid repellent and chemical resistance properties to fabrics [45]. Both PET-NP and PET-Ink samples are dipped in PDMS (1:10 curing agent:PDMS ratio, Sylgard 184) dissolved in hexane and cured in an oven at 150°C for two hours to make PET-NP/P and PET-I/P samples, respectively.

**Fig 3:**
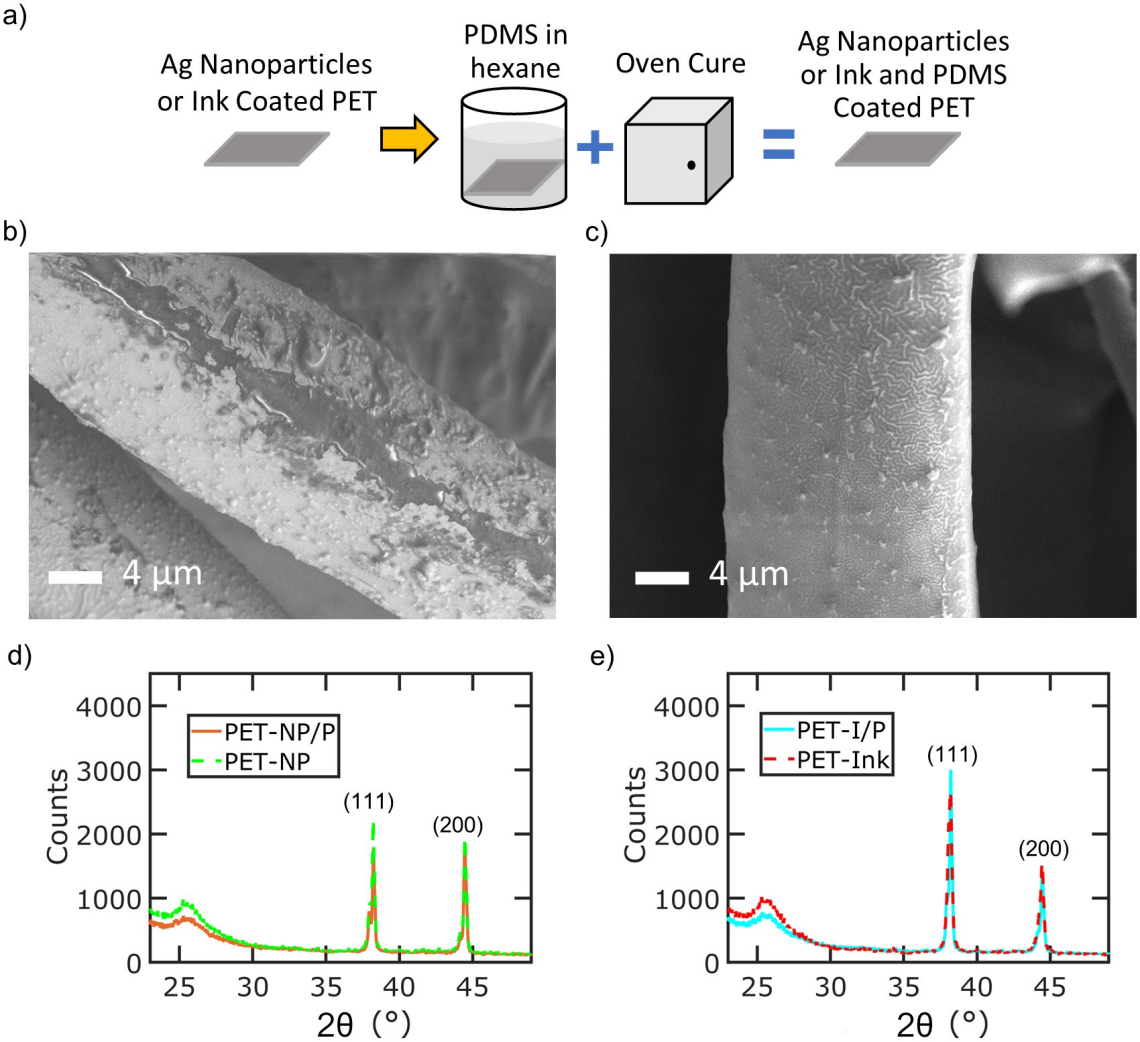
Characterization of samples with PDMS. a) Schematic of PDMS treatment for silver coated PET samples (b-c) SEM images of treated fiber of b) PET-NP/P and c) PET-I/P samples. d-e) XRD patterns of fabric samples before and after PDMS coating for d) PET-NP/P with PET-NP and e) PET-I/P with PET-Ink samples.

Fig 3 shows the characterization of PET-NP/P and PET-I/P samples. A schematic of the PDMS treatment process is depicted in Fig 3a. SEM imaging shows the physical morphology of microfibers for PET-NP/P (Fig 3b) and PET-I/P (Fig 3c) samples. The PDMS layer can be observed on the microfibers for both treatments. The PDMS layer is observed to be less than 1 μm in thickness from SEM. The PDMS coating appears rough due to the underlying morphology of the silver nanoparticles or silver ink coated fabrics. XRD characerization technique identifies the crystalline phases present on PET-NP/P (Fig 3d) and PET-I/P (Fig 3e) microfibers before and after PDMS coating. The distinct silver peaks of (111) and (200) are still present for both samples after the PDMS coating, confirming the presence of silver on the samples. The PDMS coating adds broader peaks at 2*θ* = 22 − 27° due to the amorphous polymer structure.

**Fig 4:**
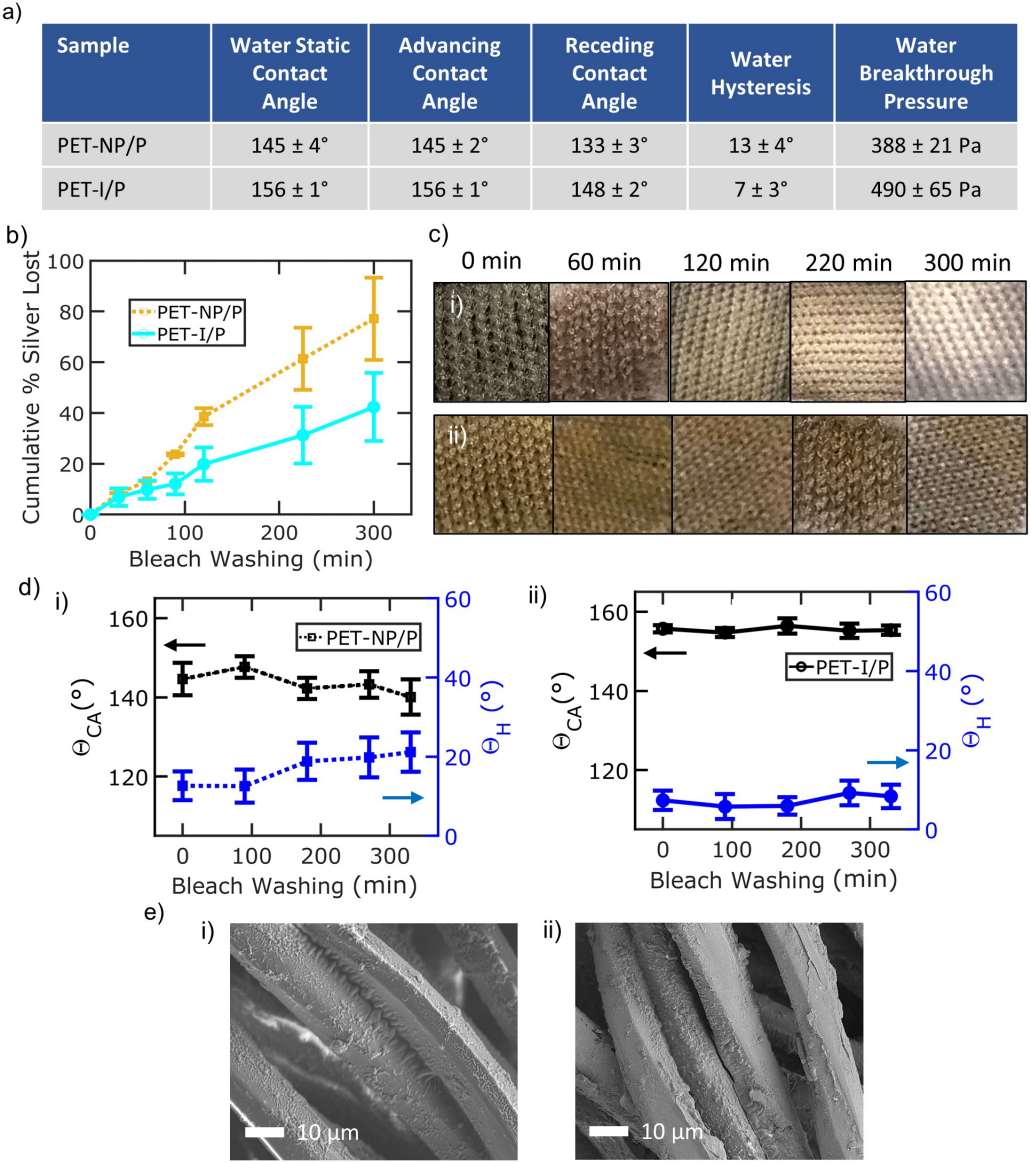
Wash Durability of samples with PDMS. a) Wetting characterization for PET-NP/P and PET-I/P samples b) Silver leaching analysis as a function of bleach washing time c) Optical images of (i) PET-NP/P and (ii) PET-I/P samples as a function of bleach washing time d) Water contact angle (black) and hysteresis (blue) as a function of ultrasonic bleach washing time for (i) PET-NP/P and (ii) PET-I/P. e) SEM images after 300 minutes of ultrasonic bleach washing time for (i) PET-NP/P and (ii) PET-I/P.

Fig 4 shows the wetting properties and bleach wash durability of PET-NP/P and PET-I/P samples. Fig 4a summarizes the wetting properties of water on silver/PDMS treated samples. The water static contact angle and hysteresis of PET-NP/P is 145 ± 4° and 13 ± 4°, respectively. The breakthrough pressure is 388 ± 21 Pa from an estimated droplet radius of 370 μm at breakthrough. The water static contact angle and hysteresis of PET-I/P is 156 ± 1° and 7 ± 3°, respectively. The breakthrough pressure is 490 ± 65 Pa from an estimated droplet radius of 294 μm at breakthrough. Representative images of the static water contact angle of treated fabrics is provided in S1 Fig in S1 File. The PDMS layer significantly improves the liquid repellency properties of the fabrics for both treatments. PET-I/P demonstrates more water repellency than PET-NP/P due to the more uniform, nanoscale roughness of the silver ink along all microfibers. In contrast, PET-NP/P samples have more exposed fabric surface area without nanoscale roughness which leads to more liquid penetration.

The cumulative percentage of silver that came off PDMS coated samples during ultrasonic bleach washing is shown in Fig 4b. The additional PDMS layer of PET-NP/P and PET-I/P samples significantly improves the retention of silver compared to PET-NP and PET-Ink samples. The PDMS layer protects the silver layer from bleach oxidation and extends the bleach washing durability of the samples. PET-NP/P samples lose more silver compared to PET-I/P samples. PET-I/P samples lose silver at a slower rate due to the stronger adhesion of silver, higher liquid repellency and higher breakthrough pressure. PET-I/P maintains about 42 ± 17% more silver than PET-NP/P after 300 ultrasonic bleach washing minutes. Fig 4c shows representative optical images of the silver loss from (i) PET-NP/P and (ii) PET-I/P as a function of bleach washing time. PET-I/P samples retain color much better than PET-NP/P samples, which are whitened from bleach washing and the removal of silver.

Fig 4d illustrates the water static contact angle and hysteresis of (i) PET-NP/P and (ii) PET-I/P samples as a function of bleach washing time. The average change in static water contact angle and hysteresis for PET-NP/P are 7 ± 2° and 9 ± 1° after 300 minutes of bleach washing. The average change in static water contact angle and hysteresis for PET-I/P are 1 ± 1° and 2 ± 1° after 300 minutes of bleach washing. The PDMS layer helps retain silver on PET samples during bleach washing; however, the PET-I/P coated samples show better performance and durability due to the stable liquid repelling properties from the conformal silver film.

The PDMS layer is structurally damaged during bleach washing, which regulates the controlled release of silver after washing. The PDMS layer for both samples appear rougher after 300 minutes of bleach washing from SEM images (Fig 4e). Loss of PDMS as well as cracking can be seen. XRD patterns of samples after bleach washing shows new distinct peaks observed at 2*θ* = 27.8°, 32.3° and 46.3° corresponding to the (111), (200) and (220) crystal planes, respectively, of silver chloride (S2 Fig in S1 File). Some of the silver is exposed to the bleach solution due to damage to the PDMS layer and reacts into silver chloride. PET is coated with PDMS only and washed to further investigate the structural integrity of the PDMS layer after bleach washing (S3 Fig in S1 File). The static contact angle increases after bleach washing, confirming the roughening of the PDMS layer. No oxidation of the PDMS layer from bleach washing is observed using FTIR analysis, so the damage to the PDMS is believed to be primarily caused by cavitation during ultrasonic agitation. These findings suggest that some damage to the PDMS layer and the silver layer occurs from ultrasonic washing with bleach.

Lastly, we measure the quantity of herpes simplex virus (HSV-1, enveloped) on fabrics, using standard plaque forming unit (PFU) assays, for samples that are unwashed, bleach washed for 100 minutes and bleach washed for 300 minutes samples. Virus assays on fabric are conducted by submerging fabrics in virus/PBS while rocking for 1 hour, then samples are removed and placed in fresh PBS. Afterwards, samples are sonicated at low power to remove viruses that adhered on the samples. The PBS liquid afterwards is used to quantify the amount of virus present on the samples. Mann-Whitney tests are utilized to offer a degree of statistical certainty that the fabric treatment reduces the amount of virus particles on the fabric compared to controls. Asterisks corresponds to the level of certainty that the treated samples are the same as the controls where one asterisk corresponds to *p* < 0.05, two asterisks for *p* < 0.01 and three asterisks for *p* < 0.001.

**Fig 5:**
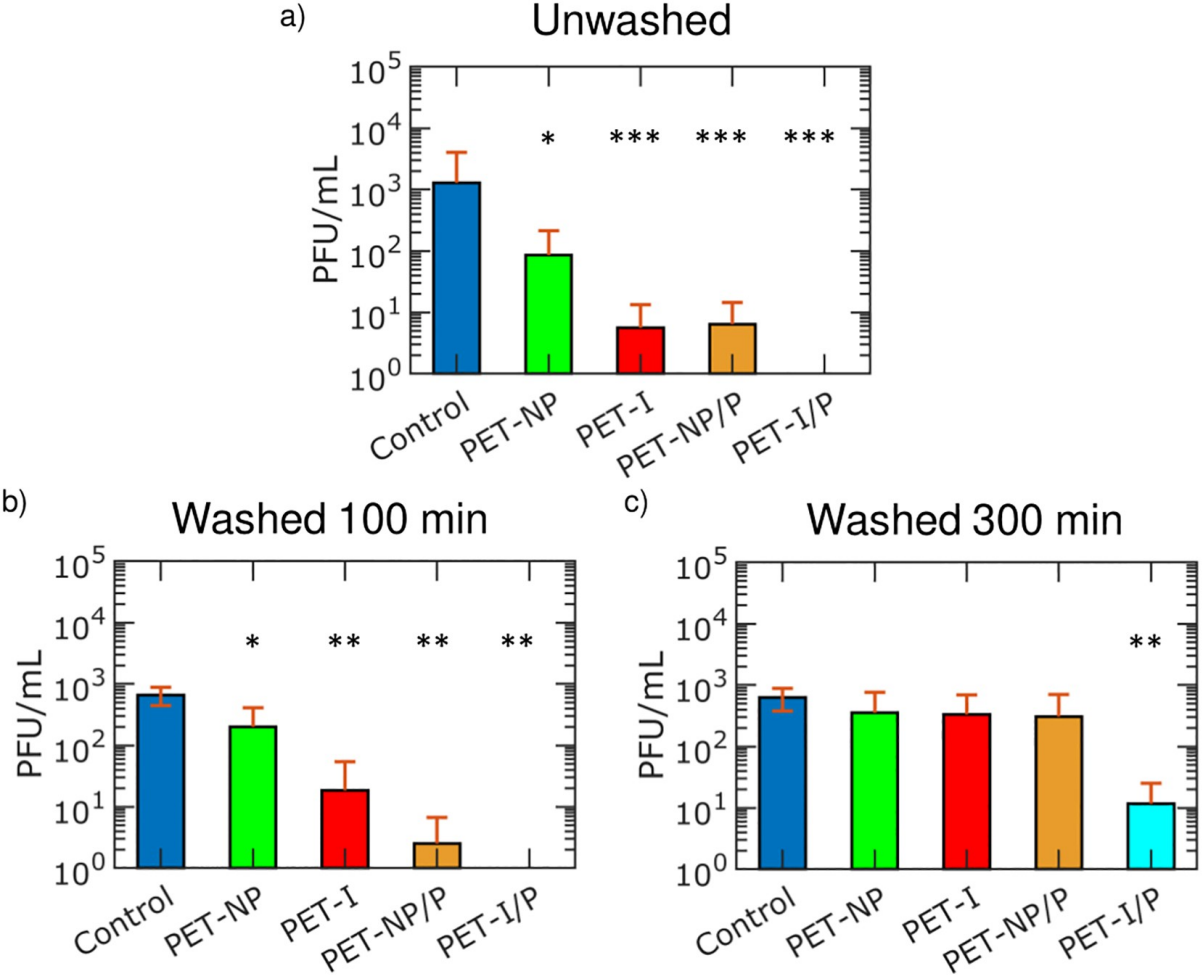
Antiviral comparison of treated fabrics before and after bleach washing. a) HSV-1 viral quantities for unwashed fabric samples b) HSV-1 viral quantities for fabric samples after 100 minutes of bleach ultrasonic washing c) HSV-1 viral quantities for fabric samples after 300 minutes of bleach ultrasonic washing.

The amount of virus on fabric samples before and after ultrasonic bleach washing is shown in Fig 5. Fig 5a shows the amount of infectious virus on unwashed fabric samples. PET-NP, PET-Ink, PET-NP/P and PET-I/P samples show a reduction of HSV-1 infection by 1.1 ± 0.6 log_10_ (92.4%, *p* < 0.05), 2.3 ± 0.8 log_10_ (99.5%, *p* < 0.001), 2.3 ± 0.8 log_10_ (99.5%, *p* < 0.001) and 2.8 ± 0.2 log_10_ (99.8%, *p* < 0.001) PFU per mL, respectively. PET-Ink samples show less infectious virus than PET-NP samples likely due to the higher antiviral activity of the silver ink in liquid. Coating the samples with PDMS reduces the amount of infectious virus observed. PDMS swells in the presence of liquid which potentially allows silver to slowly diffuse [26, 46–48]. The PDMS coated silver fabrics may release less silver than without PDMS; however, a greater reduction of infectious virus is observed with PDMS from a combination of virus inactivation and liquid repellency.

The amount of virus on fabric samples after 100 minutes of bleach washing is shown in Fig 5b. PET-NP, PET-I, PET-NP/P and PET-I/P samples show a reduction of HSV-1 by 0.6 ± 0.4 log_10_ (74.8%, *p* < 0.05), 1.6 ± 0.4 log_10_ (97.2%, *p* < 0.01), 2.4 ± 0.3 log_10_ (99.6%, *p* < 0.01) and 2.8 ± 0.1 log_10_ (99.8%, *p* < 0.01) PFU per mL, respectively. Samples without the PDMS layer may lose some functionality as significant amounts of the silver is removed from washing. Samples with the PDMS layer maintain liquid repellency and silver after 100 minutes of bleach washing due to the chemical resistance of PDMS which retains the silver layer. The changes of the performance of the samples are not statistically significant after 100 minutes of bleach washing.

Lastly, the amount of virus on fabric samples after 300 minutes of bleach washing is shown in Fig 5c. PET-NP, PET-I samples and PET-NP/P samples no longer show statistically significant reductions in HSV-1 after this extended bleach washing time from the loss of silver and liquid repellency properties. PET-I/P samples after 300 minutes of bleach washing still show a reduction of virus by 1.7 ± 0.2 log_10_ (98.2%, *p* < 0.01) PFU per mL due to the liquid repellency and virus inactivation properties. Overall, the PET-I/P treated fabrics reduce the amount of active virus, even after 300 minutes of bleach washing. The reactive silver ink coating covers the fibers more uniformly and the PDMS coating adds silver retention and liquid repellent properties for reusable, antiviral performance.

## Conclusion

There is a need for reusable, functional PPE to improve the public health safety against viral infections. This work demonstrates how reactive silver ink and a low surface energy polymer can be used to repel liquid and inactivate viruses on polyester fabric with lasting functionality under harsh ultrasonic bleach washing. The fabric treatment fully repels water before and after bleach washing. Most importantly, the fabric treatment shows reductions in enveloped virus quantities by about 2 logs compared to controls before and after bleach washing. Future work should analyze the antiviral mechanism, as well as the cytotoxicity of reactive silver ink treated fabrics before commercial use. All in all, this work demonstrates a durable, superhydrophobic, antiviral functionality on common fabric for reusable PPE applications.

## Materials and methods

### Materials

PET single knit cleanroom wipes (Anticon Gold), acetone (99.5%), methanol (99.9%) and isopropyl alcohol (99.5%) were bought from VWR. PBS (P5493), FBS (12103C), PDMS (Sylgard 184) and hexane were bought from Sigma-Aldrich. Reactive silver inks (720 series, approx. 20% silver) was provided by Electroninks. Silver nanoparticles (20 nm, 1 mg/mL in sodium citrate) were purchased from NanoComposix. Household bleach (Giant Eagle brand, SKU: 00030034936150) was purchased from Giant Eagle. Deionized water was used from a Millipore Academic A10 system with total organic carbon below 40 ppb. Herpes simplex virus were obtained from a clinical isolate of HSV-1 Mckrae and frozen at -70°C. Virus stocks in PBS were prepared with A549 human lung carcinoma cells. The virus stocks were diluted in sterile PBS to the experimental titers used.

### Sample fabrication

0.5 in. by 0.5 in. square samples were cut from PET fabric. All samples were rinsed with acetone, methanol and isopropyl alcohol and dried with nitrogen to eliminate possible contaminants. An equal amount of 0.2 mg of silver was added to fabric samples, confirmed by microscale after curing. Silver ink was added by drop casting onto untreated PET fabrics followed by oven curing for 1 hour at 120°C to make PET-Ink samples. Silver nanoparticles was added by drop casting onto untreated PET fabrics followed by oven curing for 1 hour at 120°C to make PET-NP samples. Additional PET-Ink and PET-NP samples were treated with a PDMS layer by soaking samples in PDMS (Sylgard 184) at 1:10 curing agent:PDMS ratio dissolved in hexane and curing for two hours at 150°C to make PET-I/P and PET-NP/P samples, respectively.

### Sample characterization

The physical morphologies of samples were characterized by scanning electron microscopy (SEM, Zeiss Sigma 500 VP) at 5 kV. For SEM imaging, all samples were sputter coated with 10 nm gold/palladium (80:20) using a sputter coater (Denton). The presence of silver was confirmed using X-ray diffraction (XRD, Bruker D8) locked coupled continuous scan at voltage 40 kV, current 40 mA and step size 0.04 degrees. The chemical compositions of samples were characterized by Fourier transform infrared spectroscopy (FTIR, Bruker Vertex-70LS) between wavenumber 600 and 3200 cm^−1^ using 16 scans.

Static, advancing and receding contact angle measurements were taken in ambient air at 22–25°C and 20–30% relative humidity using an optical tensiometer (Attension, 811 Theta). 5 μL droplets at 25°C for all test liquids were used for all wetting measurements. The hysteresis was tabulated for each treatment after measuring the advancing and receding contact angles during syringe-controlled water dispersion and withdrawal, respectively.

Breakthrough pressure was measured by observing the contact angle and volume while a water droplet evaporates. When the droplet transitioned from Cassie-Baxter to Wenzel state the diameter of the droplet was tabulated to calculate the breakthrough pressure.

### Durability testing

Washing cycles were performed using a Powersonic P230 Ultrasonic Cleaner (Crest) under ASTM G131–96 standards for washing materials by ultrasonic techniques. 10% bleach washing solution was prepared in a 10 mL test tube to create a highly efficient washing solution. Samples were submerged in bleach solution in Eppendorf tubes and ultrasonicated for 30 minutes at 80 W and 54°C to complete one wash cycle. Afterwards, samples were dried in ambient temperature before testing.

Silver leaching analysis was conducted using inductively coupled plasma-mass spectrophotometer (ICP-MS). Wash affluent was collected as samples were subject to bleach washing cycles. Then, samples were diluted for ICP-MS by mixing 9.8 mL of water (2% nitrate solution), 10 μL of standard and 20 μL of wash affluent and taking the weights using a microscale. The weights were used to calculate dilution factors for ICP-MS results.

### Virus assays

Virus inactivation assays were performed by mixing silver nanoparticles or silver ink with herpes virus in PBS at final silver concentration of 1 mg per mL (0.1% v/v). Solutions were vortexed and incubated for 1 hour at room temperature. After 1 hour of incubation, 500 μL of ice-cold tissue culture media containing 20% FBS was added, then vortexed and centrifuged for 1 minute in the Eppendorf centrifuge to pellet any silver. The supernatants were removed and plated with 0.1 mL of serial 10-fold dilutions in duplicate onto A549 monolayers in 24 well multiplates. The virus was absorbed for 1–3 hours after which the wells were filled with 1 mL of tissue culture media containing 0.5% methyl cellulose. Plates were incubated for 5–6 days in 5% CO_2_ and then fixed and stained with 0.5% gentian violet solution containing formalin and the plaques were quantified.

### Assays on fabrics

Fabric samples were completely submerged in 0.4 mL of virus/PBS in Eppendorf tubes with moderate shaking for 1 hour (Stoval Belly Dancer, level 5) at room temperature. After shaking, samples were gingerly rinsed once in sterile PBS and submerged in Eppendorf tubes with 0.4 mL of PBS. Then, virions attached on the surface were removed from the samples into PBS by sonication at power 3 for 10 seconds (Qsonica, Model Q55) within the tubes. The remaining PBS was used to quantify plaques.

Virus titers (PFU per mL) for herpes simplex virus were determined using standard plaque assay with A549 human lung carcinoma cells prepared in 24-well tissue culture plates. After 6–7 days incubation at 37°C in 5% CO_2_, the cells were fixed and stained with gentian violet prepared in formalin, the number of plaques per well were counted under a dissecting microscope, and viral plaque forming unit titers were calculated. Finally, Mann-Whitney U-tests were performed at different p values for all comparisons between control and treated samples.

## Supporting information

S1 File (DOCX)

S1 Graphical abstract (TIF)
