# Supplementary material for: Reactive silver inks for antiviral, repellent medical textiles with ultrasonic bleach washing durability compared to silver nanoparticles
Source: PLoS One. 2022 Sep 14;17(9):e0270718. doi: 10.1371/journal.pone.0270718 (PMC9473630; doi:10.1371/journal.pone.0270718)
Supplement: S1 File — (DOCX) [file pone.0270718.s001.docx]

Supporting Information

**Reactive silver inks for antiviral, repellent medical textiles with ultrasonic bleach washing durability compared to silver nanoparticles**

Anthony J. Galante^1^, Brady C. Pilsbury^1^, Kathleen A. Yates^2^, Melbs, LeMieux^3^, Daniel J. Bain^4^, Robert M.Q. Shanks^2^, Eric G. Romanowski^2^, and Paul W. Leu^1^*

^1^Department of Industrial Engineering, University of Pittsburgh, 3700 O'Hara, Benedum Hall, Pittsburgh, PA, 15261, United States

^2^Department of Ophthalmology, Charles T. Campbell Laboratory for Ophthalmic Microbiology, University of Pittsburgh School of Medicine, 203 Lothrop Street, Pittsburgh, PA 15213, United States

^3^Electroninks Inc, 7901 East Riverside Drive, Bldg 1, Unit 150, Austin TX 78744

^4^Department of Geology and Environmental Science, University of Pittsburgh, Pittsburgh, PA 15261, United States

* E-mail: pleu@pitt.edu


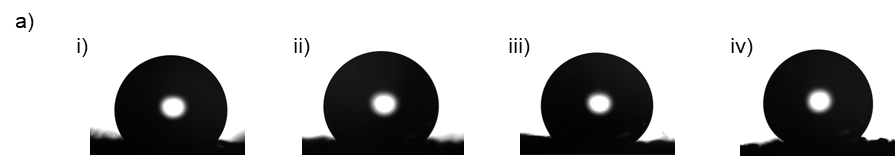


**S1 Fig.** (a) Goniometer images of static water droplets on (i) PET-NP, (ii) PET-Ink, (iii) PET-NP/P, and (iv) PET-I/P fabric samples


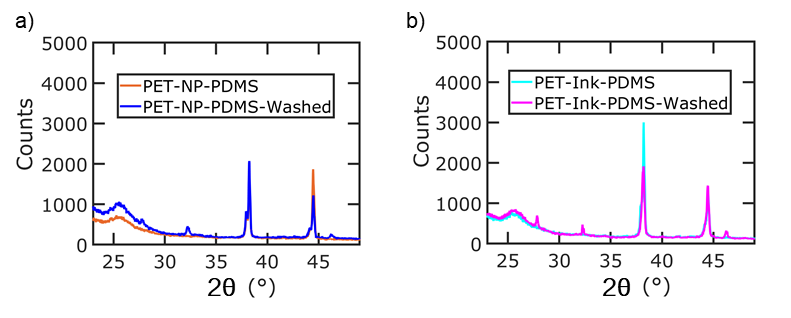


**S2 Fig.** (a-b) XRD spectra of (a) PET-NP/P and (b) PET-IP/P samples after 300 minutes of ultrasonic bleach washing.

XRD confirms the presence of silver for both samples. The XRD spectra shows new peaks after bleach washing believed to be from structural damage. XRD spectra of samples after bleach washing shows new distinct peaks observed at 2θ=27.8°, 32.3°and 46.3°corresponding to the (111), (200) and (220) crystal planes, respectively, of silver chloride. This is likely the result from some silver reacting with bleach to form silver chloride.


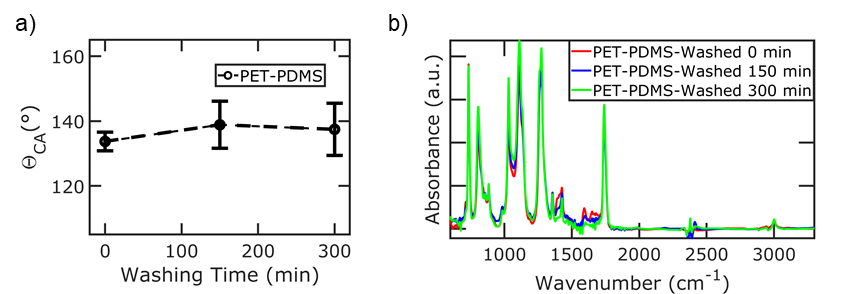


**S3 Fig.** (a) Static water contact angle of PET-PDMS samples as a function of ultrasonic bleach washing. (b)FTIR spectra of PET-PDMS samples before and after ultrasonic washing

The average static water contact angle slightly increases after bleach washing, confirming the PDMS layer is roughened from ultrasonic bleach washing. The standard deviation increases suggesting the PDMS layer is roughened non uniformly. FTIR does not show oxidation damage to the PDMS layer after washing with bleach. A small decrease in absorbance of C=C stretching around 1520-1660 cm^-1^ is observed.
