# Supplementary figures and images for: Reactive silver inks for antiviral, repellent medical textiles with ultrasonic bleach washing durability compared to silver nanoparticles
Source: PLoS One. 2022 Sep 14;17(9):e0270718. doi: 10.1371/journal.pone.0270718 (PMC9473630; doi:10.1371/journal.pone.0270718)

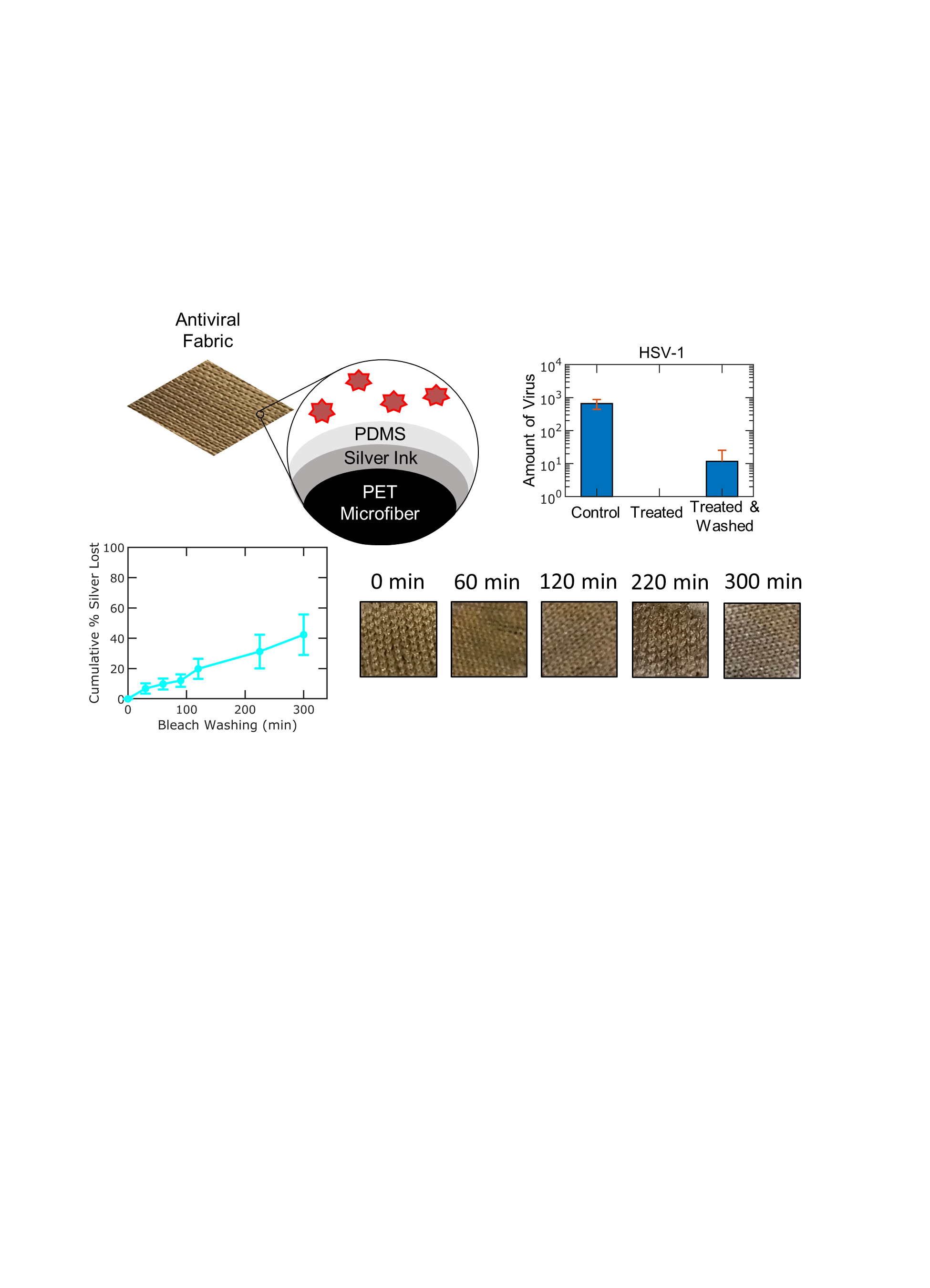

Supplement: S1 Graphical abstract — (TIF) [file pone.0270718.s002.tif]
